# Biological sex is associated with heterogeneous responses to IL-6 receptor inhibitor treatment in COVID-19—A retrospective cohort study

**DOI:** 10.1038/s41598-023-40744-y

**Published:** 2023-08-19

**Authors:** Dan F. Stein, Conor Foley, Matt Byott, Eleni Nastouli, Gareth Ambler, Nishkantha Arulkumaran

**Affiliations:** 1 Institute of Health Informatics, University College London, London, UK; 2 Advanced Pathogen Diagnostics Unit, Department of Clinical Virology, UCL Hospitals NHS Trust, London, W1T 4EU UK; 3 Department of Infection, Immunity and Inflammation, UCL Great Ormond Street Institute of Child Health, London, UK; 4 Department of Statistical Science, University College London, London, UK; 5 Bloomsbury Institute of Intensive Care Medicine, University College London, Gower St, London, WC1E 6BT UK

**Keywords:** Outcomes research, Translational research

## Abstract

COVID-19 is associated with higher inflammatory markers, illness severity and mortality in males compared to females. Differences in immune responses to COVID-19 may underpin sex- specific outcome differences. We hypothesised that anti-IL-6 receptor monoclonal antibodies are associated with heterogenous treatment effects between male and female patients. We conducted a retrospective cohort study assessing the interaction between biological sex and anti-IL-6 receptor antibody treatment with respect to hospital mortality or progression of respiratory failure. We used a Cox proportional hazards regression model to adjust for age, ethnicity, steroid use, baseline C-reactive protein, and COVID-19 variant. We included 1274 patients, of which 58% were male and 15% received anti-IL-6 receptor antibodies. There was a significant interaction between sex and anti-IL-6 receptor antibody use on progression to respiratory failure or death (*p* = 0.05). For patients who did not receive anti-IL-6 receptor antibodies, the risk of death was slightly higher in males (HR = 1.13 (0.72–1.79)), whereas in patients who did receive anti-IL-6 receptor antibodies, the risk was lower in males (HR = 0.65 (0.32–1.33)). There was a heterogenous treatment effect with anti-IL-6 receptor antibodies between males and females; with anti-IL-6 receptor antibody use having a greater benefit in preventing progression to respiratory failure or death in males (*p* = 0.05).

## Introduction

Patients with COVID-19 demonstrate a heterogeneous clinical course ranging from mildly symptomatic disease through to acute respiratory distress syndrome (ARDS) and death^1^. Early data demonstrated that COVID-19 was associated with greater severity of illness and mortality in men compared to women^2^. Whilst many lifestyle factors and co-morbid illness may be more prevalent among men, COVID-19 deaths are independently associated with advancing age, male sex, and comorbidity burden^3,4^.

The immune response between males and females is fundamentally different^5^. Dissimilarities in immune responses to COVID-19 may underpin sex- specific outcome differences. We have previously shown higher cytokine levels among males compared to females, despite similar age, viral load, degree of hypoxaemia at presentation, and requirement for organ support, consistent with an exaggerated host immune response in males^6^.

The potential benefit of several immunomodulators have been investigated in COVID-19^7^. Consistent with the literature, we and others have previously shown that IL-6 levels correlate strongly with illness severity and mortality in patients with COVID-19^8,9^. We hypothesised that anti-IL-6 receptor monoclonal antibodies are associated with heterogenous treatment effects between male and female patients. We assessed the interaction of sex and anti-IL-6 receptor monoclonal antibody treatment on clinical outcomes, including hospital survival and progression of respiratory failure in COVID-19.

## Methods

### Approval

Ethical approval was received from the London-Westminster Research Ethics Committee, the Health Research Authority and Health and Care Research Wales on 2nd July 2020 (REC reference 20/HRA/2505, IRAS ID 284088). A waiver for individual patient consent was granted. Routinely collected clinical data was curated from electronic health records including patients aged ≥ 18 years admitted to University College London Hospitals with a polymerase chain reaction- proven COVID-19 between 1st March 2020 and 30th June 2022.

All methods were performed in accordance with the relevant guidelines and regulations.

### Clinical data and definitions

Patient data were retrospectively extracted from the electronic patient record using SQL, and anonymised. Data collected included demographics, clinical data (physiological observations, laboratory tests, treatments received, organ support and outcomes.

### Inclusion and exclusion criteria

We included patients who were COVID PCR positive on throat swab with a positive test within 5 days of admission. Only patients admitted to hospital were included for analysis. Patients from the first wave (defined as until 11/12/2020, when the Alpha variant became dominant according to COG-UK data)^10^ were included if they had required supplemental oxygen during their admission. Following the first wave, patients were admitted for reasons unrelated to COVID-19 but were PCR positive for COVID on routine screening (incidental COVID-19). To ensure patients with incidental COVID-19 were not included, patients from subsequent waves were included only if they required supplemental oxygen therapy within 48h of admission and received treatment for COVID, with either steroids or another recognized COVID therapy. Patients who were transferred to UCLH from another hospital within 48 h of their initial admission with clinically diagnosed COVID were included.

Routine use of anti-IL-6 receptor antibodies (either tocilizumab and sarilumab) for the management of acute COVID-19 at our centre followed the initial announcement of the REMAP-CAP CAP study data^11^. Our criteria for use of anti-IL-6 receptor antibodies included patients admitted with COVID-19 and CRP > 75 mg/L; or patients admitted to ICU requiring respiratory support (non-invasive positive pressure ventilation, high flow oxygen therapy, or mechanical ventilation) within 24 h of admission. We avoided the use of anti-IL-6 receptor antibodies in patients with known or suspicion of bacterial infection, raised liver function tests (ALT or AST > 5 × upper limit), platelet count < 50 × 10^9^, or neutrophil count < 2 × 10^9^.

### Primary and secondary outcomes

The primary outcome was hospital mortality. The secondary outcome was a composite of mortality and requirement for increased respiratory support. As the treating clinician may have deemed some patients unsuitable for escalation to invasive mechanical ventilation, a composite of mortality or requirement for increased respiratory support (respiratory deterioration) was taken as a secondary outcome. We previously described a strong correlation between serum IL-6 and CRP; IL-6 being a key regulator of C-reactive protein (CRP) production^9^. We therefore also investigated the association between use of anti-IL-6 receptor monoclonal antibodies and trajectory of CRP. Patients who died within 48 h of hospital admission were excluded.

### Data validation

Data was obtained via an automatic data pull from the hospital electronic health record database, and subsequently validated against independently collected datasets within the hospital. Two months of positive COVID-19 PCR tests were manually validated. Data were also cross validated against an independent virology dataset, manually collected by that department, with this dataset within 3% of the manually validated set.

### Statistical analysis

Analysis was performed using pseudo-anonymised data, and all analysis was performed within the hospital information technology systems and servers. Data analysis was performed in R 4.0.0. Groups were compared at baseline using chi-square, Mann–Whitney U and ANOVA tests (Table 1). Kaplan–Meier plots were produced to visualise survival trajectories, and unadjusted survival was compared between groups using a log-rank test. We used a Cox proportional hazards regression model to adjust for confounders including age, ethnicity, baseline illness severity (using CRP as a surrogate), and variant of COVID-19.

**Table 1:** Demographics, treatment, and outcome. Continuous data are presented as median (interquartile range) and categorical data presented as number (%). (JAK: Janus kinase; HFNO: high flow nasal oxygen, NIV: Non- invasive ventilation).

	Total (n = 1274)	Male No IL-6 inhibitor (n = 627)	Male IL-6 inhibitor (n = 116)	Female No IL-6 inhibitor (n = 458)	Female IL-6 inhibitor (n = 73)	*p* value
Median age in years (IQR)	64.0 (51.2–76.1)	64.1 (51.0–76.4)	58.3 (49.0–69.8)	65.6 (52.1–78.3)	61.5 (51.2–72.6)	0.02
Ethnicity (%)						
White	562 (44.1)	283 (45.1)	38 (32.8)	208 (45.4)	33 (45.2)	0.0002
Black	145 (11.4)	53 (8.5)	15 (12.9)	62 (13.5)	15 (20.5)	
Asian	150 (11.8)	78 (12.4)	7 (6.0)	56 (12.2)	9 (12.3)	
Other	150 (11.8)	70 (11.2)	18 (15.5)	54 (11.8)	8 (11.0)	
Unknown	267 (21.0)	143 (22.8)	38 (32.8)	78 (17.0)	8 (11.0)	
Variant (%)						
Wildtype	401 (31.5)	247 (39.4)	4 (3.4)	150 (33.8)	0 (0)	< 0.0001
Alpha	522 (41.0)	275 (43.9)	30 (25.9)	200 (43.7)	17 (23.3)	
Delta	269 (21.1)	70 (11.2)	73 (62.9)	73 (15.9)	53 (72.6)	
Omicron	82 (6.4)	35 (5.6)	9 (7.8)	35 (7.6)	3 (4.1)	
Median admission CRP (mg/L) (IQR)	86.5 (42.8–154.7)	87.1 (43.0–162)	131 (85.4–183)	64.9 (31.2–124)	127 (98.0–199)	< 0.0001
Median admission lymphocyte (10^6^/mL) (IQR)	0.93 (0.64–1.30)	0.91 (0.63–1.30)	0.83 (0.62–1.03)	0.99 (0.71–1.37)	0.91 (0.52–1.28)	0.30
Steroid use (%)	1019 (80.0)	472 (75.3)	115 (99.1)	359 (78.4)	73 (100)	< 0.0001
Antiviral drug use (%)						
Remdesivir	299 (23.3)	143 (22.8)	37 (31.9)	98 (21.4)	21 (28.8)	0.073
JAK inhibitor	2 (0.16)	0 (0.0)	0 (0.0)	2 (0.44)	0 (0.0)	0.31
Admission respiratory support (%)						
Intubated	71 (5.6)	37 (5.9)	2 (1.7)	28 (6.1)	4 (5.5)	< 0.0001
HFNO	53 (4.2)	17 (2.7)	16 (13.8)	14 (3.1)	6 (8.2)	
NIV	267 (21.0)	135 (21.5)	44 (37.9)	62 (13.5)	26 (35.6)	
Supplementary O_2_	883 (69.3)	438 (69.9)	54 (46.6)	354 (77.3)	37 (50.7)	
Highest level of respiratory support or death (%)						
Died	290 (22.9)	162 (25.8)	18 (15.5)	92 (20.1)	18 (24.7)	< 0.0001
Intubated	69 (5.4)	32 (5.1)	7 (6.0)	26 (5.7)	4 (5.5)	
HFNO	52 (4.1)	16 (2.6)	13 (11.2)	14 (3.1)	9 (12.3)	
NIV	202 (15.9)	93 (14.8)	37 (31.9)	50 (10.9)	22 (30.1)	
Supplementary O_2_	661 (51.9)	324 (51.7)	41(35.3)	276 (60.3)	20 (27.4)	

To investigate the association between CRP trajectory and other factors such as such as sex, ethnicity and variant, we used a mixed effects model with repeated measurements of CRP over the first 14 days of admission used. Inter-patient variability was modelled using random intercepts and slopes whilst fixed effects were used for age, sex and anti-IL-6 receptor monoclonal antibody use. Interaction terms between the variables of interest and day of admission were used to investigate whether CRP trajectory over time differs depending on these variables. CRP trajectory was modelled as linear, and plots were produced to validate this. Statistical significance was set at 0.05 for all analysis.

## Results

### Demographics

A total of 1274 patients were included (Table 1), after excluding 16 patients who died within 48 h of hospital admission. Of the 1274 patients, the median age was 64 (51–76) years, and 58% were male. A total of 189 patients (14.8%) received anti-IL-6 receptor monoclonal antibodies, which included 116 (15.6%) male patients and 73 (13.7%) female patients. Majority (44%) of patients were White, with similar proportions of Black (11.4%), Asian (11.8%) and other (11.8%) ethnicities. Ethnicity was unrecorded in 21.0% of cases. The alpha variant (41.0%) accounted for majority of cases, followed by the Wild type (31.5%) and delta variant (21.1%). Admission CRP was 86.5 (42.8–154.7) mg/L and lymphocyte count was 0.93 (0.64–1.30) × 10^6^/mL.

Approximately one quarter (23.3%) of patients received Remdesivir, and only two patients received a JAK inhibitor. All patients apart from one (188/189, 99.5%) receiving anti-IL-6 receptor monoclonal antibodies were also prescribed steroids. On hospital admission, most patients (69%) required supplemental oxygen alone, with only 5.6% of patients requiring mechanical ventilation. Overall hospital mortality was 22.9%.

## Effect of IL-6 inhibitors on CRP

CRP on admission to hospital was similar between male (median 131, IQR: 85.4–183) and female (median 127, IQR: 98.0–199) patients who received anti-IL-6 receptor monoclonal antibodies (*p* = 0.89). However, among patients who did not receive anti-IL-6 receptor monoclonal antibodies, male patients (median 87.1, IQR: 43.0–162) had a higher admission CRP than female patients (median 64.9, IQR: 31.2–124) (*p* < 0.001). CRP on admission to hospital was higher in patients who received anti-IL-6 receptor monoclonal antibodies compared to those who did not, among both male (*p* < 0.001) and female (< 0.001) patients.

**Figure 1:**
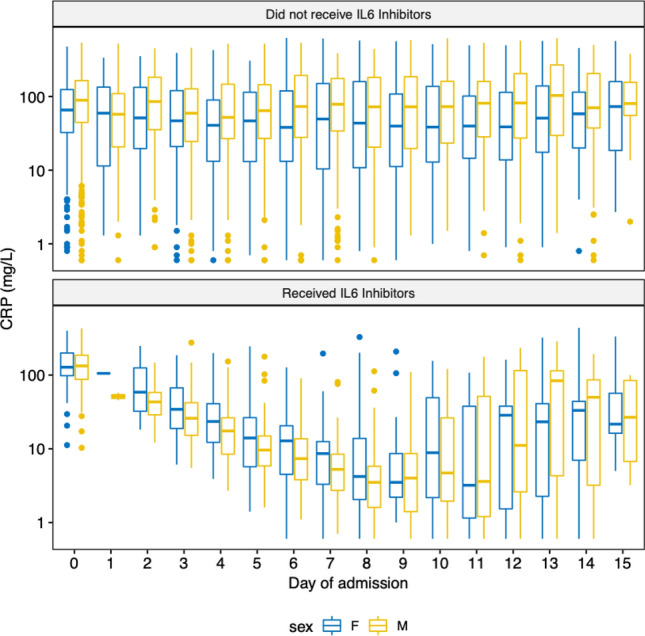
Box and whisker plots of C-reactive protein (CRP) by day of admission in male and female patients who did or did not receive IL- 6 inhibitors. Note logarithmic y-axis.

After adjustment for age and sex, anti-IL-6 receptor monoclonal antibody use was associated with a greater rate of fall in CRP change over time compared to patients not treated with anti-IL-6 receptor monoclonal antibodies (*p* < 0.001) (Fig. 1). However, there was no significant interaction between the rate of fall of CRP and either sex alone or sex and anti-IL-6 receptor monoclonal antibody use (Supplementary Table 1).

## Effect of IL-6 inhibitors on mortality

**Figure 2:**
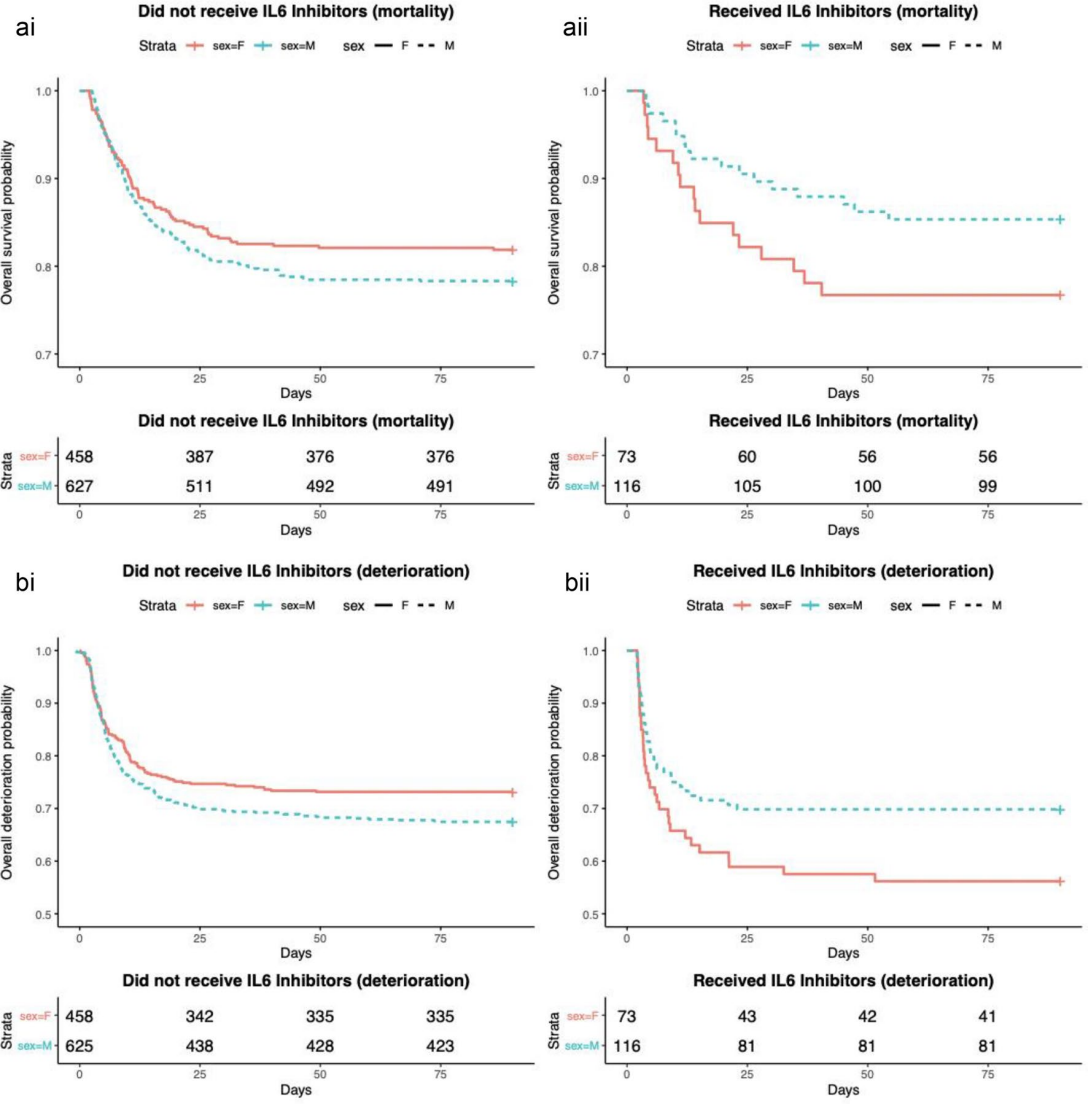
Kaplan–Meier plots for (**a**) hospital mortality and (**b**) composite of progression of respiratory support or death in male and female patients who did or did not receive IL-6 inhibitors. The unadjusted hospital mortality was not significantly different between males and females who (ai) did not (*p* = 0.17) or (aii) did (*p* = 0.12) receive an anti-IL-6 receptor monoclonal antibody. (bi) Among patients who did not receive anti-IL-6 receptor monoclonal antibodies, there was an increased unadjusted risk of progression of respiratory failure or death among males compared to female patients (*p* = 0.058), although not statistically significant. (bii). Among patients who received anti-IL-6 receptor monoclonal antibodies, there was an increased unadjusted risk of progression of respiratory failure or death among females compared to male patients (*p* = 0.066) although not statistically significant. All *p* values were calculated using log-rank test.

The unadjusted hospital mortality was not significantly different between males and females who did (*p* = 0.12) and did not (*p* = 0.17) receive an anti-IL-6 receptor monoclonal antibody (Fig. 2a), although the Kaplan–Meier plots do suggest a trend towards difference. Following adjustment for age, baseline CRP, ethnicity, steroid use, and COVID-19 variant, there was evidence that the effect of treatment with anti-IL-6 receptor monoclonal antibodies differs between males and females (*p* = 0.14), although this was not statistically significant. Among patients who did not receive anti-IL-6 receptor monoclonal antibodies, we observed increased mortality for male sex, although this was not statistically significant (HR = 1.56; CI (0.89–2.71)). By contrast, among patients who did receive treatment with anti-IL-6 receptor monoclonal antibodies, we observed decreased mortality for male sex (HR = 0.89; CI (0.36–2.24)). There was no effect of sex on response to treatment with steroids (Supplementary Figure 1).

## Effect of IL-6 inhibitors on composite of progression of respiratory failure and mortality

There is a significant difference in baseline respiratory support between patients who were treated with and without anti-IL-6 receptor monoclonal antibodies (*p* < 0.001) (Table 1). Fifty-three percent of males who were administered anti-IL-6 receptor monoclonal antibodies required more than supplemental O_2_ support on hospital admission, compared to 30% males who did not receive anti-IL-6 receptor monoclonal antibodies. Similarly, 49% of females who were administered IL-6 inhibitor required more than supplemental O_2_ support, compared to 23% females who did not receive IL-6 inhibitors.

Among patients who did not receive anti-IL-6 receptor monoclonal antibodies, there was an increased unadjusted risk of progression of respiratory failure or death among males compared to female patients; although this did not quite reach statistical significance (*p* = 0.058). The reverse was true among patients who received anti-IL-6 receptor monoclonal antibodies. There was an increased unadjusted risk of progression of respiratory failure or death among females compared to male patients; although this did not reach statistical significance (*p* = 0.066) (Fig. 2b).

Following adjustment for age, baseline CRP, ethnicity, and COVID-19 variant, there was a significant difference in progression to respiratory failure or death in response to treatment between males and females (*p* = 0.05). Among patients who did not receive IL-6 inhibitors, males were at increased risk of progression to respiratory failure or death compared to females (HR = 1.13; CI (0.72–1.79)). In contrast, among patients who did receive IL-6 inhibitors, males were at lower risk of progression to respiratory failure or death compared to females (HR = 0.65; CI (0.32–1.33)). This differential effect of treatment on the composite of progression of respiratory failure or death between males and females was not seen with treatment with steroids (*p* = 0.82) (Supplementary Figure 1).

## Discussion

We demonstrate heterogeneity in response to treatment with anti-IL-6 receptor monoclonal antibodies between males and females with COVID-19. Sex had a significant interaction with anti-IL-6 receptor monoclonal antibody use on progression to respiratory failure or death; with male patients having a greater benefit associated with anti-IL-6 receptor monoclonal antibody use. Although the association between greater severity of illness and mortality in men compared to women in COVID-19 is well- described, there is paucity of data on the impact of COVID-19 treatments on clinical responses between male and female patients.

The immune response between males and females is fundamentally different. As a large number of genes related to immune functions are located on the X chromosome, X-linked mosaicism confers a highly polymorphic gene expression program that allows women to respond with a more expanded immune repertoire as compared with men^5^. Differences in immune response between males and females extend from responses to bacterial infections to viral vaccines^12,13^.

Despite a similar incidence of COVID-19 diagnoses in males and females in the community, the case fatality rate among males is significantly higher^14,15^. Biological mechanisms underpinning these observations have been investigated in an attempt to better understand the pathophysiology of COVID-19^15^. Several differences in the immune response between males and females have been described including higher pro-inflammatory innate immunity chemokines and cytokines in male patients^6,16^. Greater expression of virus entry factors (Angiotensin-converting enzyme 2 (ACE2) and accessory proteases (TMPRSS2 and CTSL) in airway secretory cells and alveolar type 2 cells in males may explain the greater cytokine levels in male patients^17^. In addition to differences in cytokine levels between sexes, a poor T cell response is associated with worse disease outcome in male patients, but not in female patients^16^.

Despite stark differences in the proportions of males compared to females affected by COVID-19 in early reports^2^, few clinical trials in COVID-19 reported outcomes by sex. Where reported, differences in responses to treatment with immunosuppression have been described in some instances^18^. The National Institutes of Health (NIH) announced policies in May 2014 that “require applicants to report their plans for the balance of male and female cells and animals in preclinical studies in all future applications, unless sex-specific inclusion is unwarranted, based on rigorously defined exceptions^19^.” Inclusion of sex as a research variable has the potential promote discovery of disease mechanisms and effective treatments^20^. As an example, the efficacy of novel cancer immunotherapies is influenced by sex differences in genetic and hormone-mediated immune responses^21^.

We describe novel findings on the different response to treatment with anti-IL-6 receptor monoclonal antibodies between males and females with COVID-19. However, we were unable to ascertain data on COVID-19 vaccination status, and co-morbid illness. Additionally, most patients received steroids and this was an inclusion criterion after the first wave, potentially confounding comparisons between patients who did and did not receive steroid treatment. We did not find any significant interactions between sex, anti-IL-6 receptor monoclonal antibody use and fall in CRP, possibly because we were unable to censor for patients who were discharged home, or those who died in hospital. Similarly, we did not find any significant interactions between sex, anti-IL-6 receptor monoclonal antibody use, and mortality. However, a composite of mortality or requirement for increased respiratory support (respiratory deterioration) was a clinically relevant outcome that captures the beneficial effect of anti-IL-6 receptor monoclonal antibody use. Any differences in age between patient groups have been adjusted for in the multivariate analysis, and therefore reflected in the final outcome(s).

Lack of differences in mortality to treatment with anti-IL-6R antibodies in COVID-19 by biological sex has been described in a recent meta-analysis of RCTs^22^. This survival data are consistent with ours, although we saw a non- statistically significant trend to improved survival in female patients treated with anti-IL-6R antibodies. However, the effect of anti-IL-6R antibodies on the progression of respiratory failure or mechanical ventilation (either as an individual outcome or as a composite outcome with death) was not reported in this meta-analysis. Differences in reported outcomes between studies are multiple, including differences in patient populations, criteria for treatment with treatment with anti-IL-6R antibodies, and unmeasured confounders not adjusted for in our analyses. Crucially, our analysis included patients who did not meet the requirement for treatment with anti-IL-6R antibodies whereas the RCTs only report outcomes in patients eligible for treatment with anti-IL-6R antibodies.

The Recovery study reported a greater treatment benefit of dexamethasone on 28-day mortality in male compared to female patients with COVID-19^18^. This supports our finding, that an anti-inflammatory treatment in COVID-19 is associated with a different response between males and females; although we did not find any interaction between sex, steroid use, and hospital mortality. However, our sample size was relatively small, particularly with regards to assessing the effect of interactions between covariates, and steroid use was an inclusion criterion from December 2020 our data. Finally, as with all retrospective analyses, we cannot correct for residual confounding; any associations cannot be interpreted as causal.

In summary, we demonstrate that sex had a significant interaction with anti-IL-6 receptor monoclonal antibody use on progression to respiratory failure or death; with male patients having a greater benefit associated with anti-IL-6 receptor monoclonal antibody use. Differences in immune response between males and females are well recognised^23^. However, the lack of sex-based analyses in both clinical and pre-clinical studies persists. The disproportionate effect of COVID-19 on illness severity in males compared to females highlights the importance of sex-based analyses of therapeutic interventions. Greater appreciation of this among scientists, peer-reviewers, and scientific journals will facilitate better understanding of mechanisms of disease, and could be a further step towards personalised medicine.

### Supplementary Information

Supplementary Information.

## Data Availability

Anonymised data can be available under reasonable request. The corresponding author should be contacted.
