# Supplementary material for: Biological sex is associated with heterogeneous responses to IL-6 receptor inhibitor treatment in COVID-19—A retrospective cohort study
Source: Sci Rep. 2023 Aug 19;13:13504. doi: 10.1038/s41598-023-40744-y (PMC10439929; doi:10.1038/s41598-023-40744-y)
Supplement: Supplementary file 1 — Supplementary Information. [file 41598_2023_40744_MOESM1_ESM.docx]

**Supplementary data**

aii

bi

ai

bii

ai

**Supplementary Figure 1**: Kaplan-Meier plots for (a) hospital mortality and (b) composite of progression of respiratory support or death in male and female patients who did or did not receive steroids. The unadjusted hospital mortality was not significantly different between males and females who (ai) did not (p=0.75) or (aii) did (p=0.53) receive steroids. (bi) The unadjusted risk of progression of respiratory failure or death was not significantly different between males and females who (bi) did not (p=0.98) or (bii) did (p=0.25) receive steroids. All p-values were calculated using log-rank test.

**Supplementary Table 1:** Results from mixed effects model. The model formula used was as follows: CRP ~ b0 + b1*DAY + b2*AGE + b3*MALE + b4*IL6 + b5*DAY*AGE + b6*DAY*MALE + b7*MALE*IL6 + b8*DAY*IL6 + b9*DAY*MALE*IL6. In this model DAY denotes day of admission, MALE denotes male sex, AGE denotes biological age in years, IL6 was a binary variable denoting whether or not the patient was treated with an anti-IL6 receptor monoclonal antibody. The model used has random intercepts and slopes for DAY.

| Variable | Effect Estimate | P-Value | 95% CI (lower bound) | 95% CI (upper bound) |
| --- | --- | --- | --- | --- |
| DAY | -5.07 | 0.011 | -9.00 | -1.11 |
| AGE | 0.50 | 0.0005 | 0.23 | 0.78 |
| MALE | 21.2 | <0.0001 | 10.8 | 31.5 |
| IL6 | 20.9 | 0.04 | 0.75 | 41.1 |
| AGE*DAY | 0.03 | 0.34 | -0.03 | 0.08 |
| MALE*DAY | 0.30 | 0.77 | 1.67 | 2.26 |
| MALE*IL6 | -24.1 | 0.07 | -50.1 | 1.82 |
| IL6*DAY | -7.96 | <0.0001 | -11.5 | -4.4 |
| MALE*IL6*DAY | 0.33 | 0.88 | -4.11 | 4.78 |
